# Synthesis and Antimicrobial Evaluation of Some New Oxadiazoles Derived from Phenylpropionohydrazides

**DOI:** 10.3390/molecules14051898

**Published:** 2009-05-20

**Authors:** Neeraj Kumar Fuloria, Vijender Singh, Mohammad Shaharyar, Mohammad Ali

**Affiliations:** 1 Department of Pharmacy, Rameesh Institute of Vocational and Technical Education, 3-Knowledge Park-1, Kasna road, Greater NOIDA, India; E-mail: mahalwal_vs@yahoo.com (V.S.); 2 Department of Pharmaceutical Chemistry, Faculty of Pharmacy, Jamia Hamdard, Hamdard Nagar, New Delhi-110062, India; E-mail: yarmsy@rediffmail.com (M.S.); 3 Department of Pharmacognosy & Phytochemistry, Faculty of Pharmacy, Jamia Hamdard, Hamdard Nagar, New Delhi-110062, India

**Keywords:** phenylpropionohydrazide, imines, oxadiazoles, antibacterial, antifungal

## Abstract

In this study a series of new 1-(2-aryl-5-phenethyl-1,3,4-oxadiazol-3(2H)-yl)ethanones **2a-e** was synthesized by the cyclization of imines **1a-e** using acetic anhydride. The products were evaluated for anti-bacterial and anti-fungal activity. Among the newly synthesized compounds, *1-(2-(4-(dimethylamino)phenyl)-5-phenethyl-1,3,4-oxadiazol-3(2H)-yl)ethanone* (**2a**) and *1-(2-(4-chlorophenyl)-5-phenethyl-1,3,4-oxadiazol-3(2H)-yl)ethanone* (**2b**) were found to possess maximum activity against the tested strains of *S. aureus* and *P. aeruginosa.* It was concluded that *para*-substitution enhances the activity of synthesized oxadiazoles.

## 1. Introduction

It is an established fact that oxadiazoles, imines and propanoates exhibits antimitotic [1], antikinetoplastid [2], antitussive [3], hybrid COX-2 inhibitor/nitric oxide donor [4], antimycotic [5], anti-inflammatory [6], analgesic [7], antimicrobial and anticonvulsant [8,9,10,11,12] activities. Moreover esters and hydrazides can be converted into imines, which are precursor for oxadiazoles [8,9,10,11,12]. The literature has reported different biological activities and method of synthesis for oxadiazoles [1,2,3,4,5,6,7,8,9,10,11,12]. Hence an attempt was made to convert some *N*-(substituted benzylidene)-3-phenylpropionohydrazides into novel 1-(2-aryl-5-phenethyl-1,3,4-oxadiazol-3(2H)-yl)ethanones. The novel compounds were characterized and further investigated for anti-bacterial and anti-fungal activities.

## 2. Results and Discussion 

### 2.1. Chemistry

**Scheme 1:**
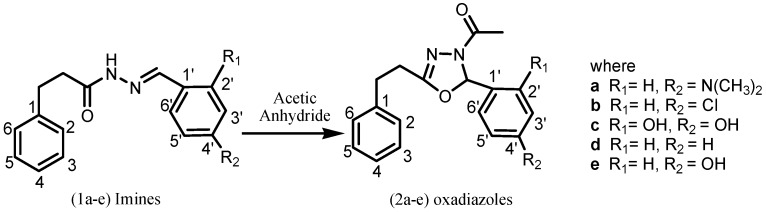
Synthesis of Oxadiazoles **2a-e**.

The treatment of *N*-(substituted benzylidene)-3-phenylpropionohydrazides **1a-e**, with acetic anhydride yielded 1-(2-aryl-5-phenethyl-1,3,4-oxadiazol-3(2H)-yl)ethanones **2a-e** (Scheme 1).

**Figure 1:**
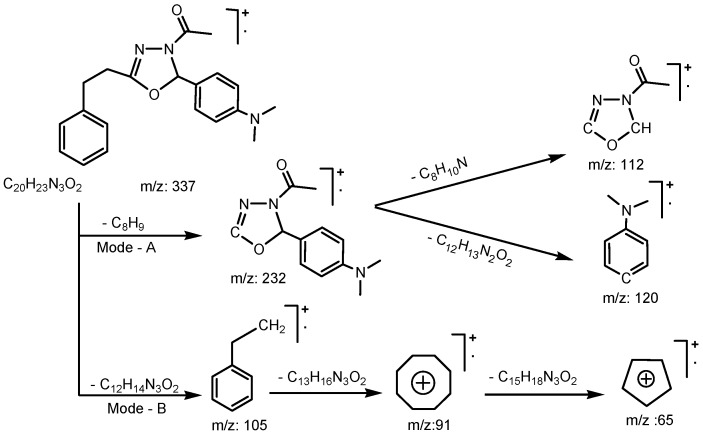
fragmentation pattern of oxadiazole **2a**.

The carbonylamino and imino groups in compounds **1a-e**, were found to cyclize to form oxadiazole rings when reacted with acetic anhydride. The assigned structures, molecular formulae and the anomeric configuration of the newly synthesized oxadiazoles **2a-e** were further confirmed and supported by mass, ^1^H-NMR and IR spectrometry. The fragmentation pattern of compound **2a** due to absence of C_8_H_9_, C_12_H_13_N_2_O_2_, C_16_H_19_N, C_12_H_14_N_3_O_2_, C_13_H_16_N_3_O_2_, C_15_H_18_N_3_O_2_ groups given in Figure 1, as an example further supported in identification of molecular structures of compounds **2a-e**.

The absence of specific group fragments in the mass spectra of compounds **2b-e** (C_8_H_9_, C_12_H_13_N_2_O_2_, C_14_H_13_Cl, C_10_H_8_ClN_2_O_2_, C_11_H_10_ClN_2_O_2_, C_13_H_12_ClN_2_O_2_ in the case of **2b;** C_8_H_9_, C_12_H_13_N_2_O_2_, -C_14_H_14_O_2_, C_10_H_9_N_2_O_4_, C_11_H_11_N_2_O_4_, C_13_H_13_N_2_O_4_ in **2c;** C_8_H_9_, C_12_H_13_N_2_O_2_, C_14_H_14_, C_10_H_9_N_2_O_2_, C_11_H_11_N_2_O_2_, C_13_H_13_N_2_O_2_ **2d**; and C_8_H_9_, C_12_H_13_N_2_O_2_, C_14_H_14_O, C_10_H_9_N_2_O_3_, C_11_H_11_N_2_O_3_, C_13_H_13_N_2_O_3_ in **2e**) was a key to establish their molecular structures. The purity of the compounds was checked by melting point, TLC and elemental analysis results, which were within ± 0.4% of the theoretical values.

### 2.2. Biological activity

The newly synthesized compounds **2a-e** were screened for antibacterial activity against freshly cultured strains of *S. aureus* (SA) and *P. aeruginosa* (PA) using sterile nutrient agar media and for antifungal activity against freshly cultured strains of *C. albicans* (CA) and *A. flavus* (AF) using sterile sabouraud’s agar medium by the disk diffusion method at a concentration of 2 mg per mL. using DMF as solvent. The results were recorded in duplicate using ampicillin and fluconazole at a concentration of 1 mg per mL as standards.

Among newly synthesized derivatives, compounds **2a** and **2b** were found to be equipotent to ampicillin when tested against the strains of *S. aureus*, and *P. aeruginosa*, whereas some of the newly synthesized compounds like **2a**, **2d** and **2e** were found to possess good antibacterial and antifungal activity when tested against *S. aureus*, *P. aeruginosa*, *C. albicans* and *A. flavus* (Table 1). 

## 3. Experimental Section

### 3.1. General

Melting points of newly synthesized compounds were determined using Thomas Hoover apparatus. IR spectra were recorded (in KBr) on a Bruker PCIR, ^1^H-NMR on Bruker, DPX 300 and mass spectra on MASPEC (MSW/9629). Purity of synthesized compounds was checked by TLC aluminium sheets – silica gel 60 F254 (0.2 mm).

### 3.2. General procedure for the synthesis of 1-(2-aryl-5-phenethyl-1,3,4-oxadiazol-3(2H)-yl)ethanones *(**2a-e**)*

A mixture of compound **1a-e** (0.01 mol) derived from 3-phenyl propane hydrazide was refluxed with acetic anhydride (0.01 mol) for 12 hours in the presence of zinc chloride. The product formed was isolated by filtration and recrystallized from methanol to yield compounds **2a-e.**

*1-(2-(4-(Dimethylamino)phenyl)-5-phenethyl-1,3,4-oxadiazol-3(2H)-yl)ethanone* (**2a**): Pale yellow crystals; Yield 65.8%; mp 225-226ºC; ^1^H-NMR δ (ppm): 2.04 (3H, s, -CO-CH_3_), 2.32 (2H, t, 6.9Hz, -CH_2_-C-O-), 2.65 (2H, t, 6.9Hz, Ar-CH_2_), 2.89 (6H, s, -N(CH_3_)_2_), 6.52 (2H, d, 8.1Hz, Ar-H3′ & 5′), 6.65 (1H, s, -N-CH-Ar′), 7.01 (2H, d, 8.2Hz, Ar′-H2′ & 6′), 7.18-7.31 (5H, m, Ar-H2, 3, 4, 5 & 6); FT-IR: 2924 (C-H of CH_2_), 1688 (C=O), 1611 (C=N), 1259 (C-O-C) cm^-1^; Anal. Calcd. for C_20_H_23_N_3_O_2_ (337.42): C: 71.19, H: 6.87, N: 12.45. found: C: 71.16, H: 6.85, N: 12.43; MS: m/z: 337 (M^+^), 232 (base peak), 120, 112, 105, 91, 65.

*1-(2-(4-Chlorophenyl)-5-phenethyl-1,3,4-oxadiazol-3(2H)-yl)ethanone* (**2b**): White crystals; Yield 64.9%; mp 212-213 °C; ^1^H-NMR δ (ppm): 2.11 (3H, s, -CO-CH_3_), 2.38 (2H, t, 6.5Hz, -CH_2_-C-O-), 2.72 (2H, t, 6.6Hz, Ar-CH_2_), 6.64 (1H, s, -N-CH-Ar′), 7.14 (2H, d, 8.3Hz, Ar′-H2′ & 6′), 7.20 (2H, d, 8.1Hz, Ar′-H3′ & 5′), 7.24-7.37 (5H, m, Ar-H2, 3, 4, 5 & 6); FT-IR: 1608 (C=N), 2928 (C-H of CH_2_), 1681 (C=O), 1256 (C-O-C) cm^-1^; Anal. Calcd. for C_18_H_17_N_2_O_2_Cl (328.79): C: 65.75, H: 5.21, N: 8.52. Found: C: 65.72, H: 5.20, N: 8.50; MS: m/z 328 (M^+^), 223 (base peak), 112, 111, 105, 91, 65.

*1-(2-(2,4-Dihydroxyphenyl)-5-phenethyl-1,3,4-oxadiazol-3(2H)-yl) ethanone* (**2c**): Yellow brown crystals; Yield 59.2%; mp 219-220 °C; ^1^H-NMR δ (ppm): 2.07 (3H, s, -CO-CH_3_), 2.32 (2H, t, 6.8Hz, -CH_2_-C-O-), 2.62 (2H, t, 6.8Hz, Ar-CH_2_), 5.22 (1H, s, 4-OH), 5.28 (1H, s, 2-OH), 6.14 (1H, d, 2.6 Hz, Ar′-H3′), 6.28 (1H, dd, 2.8, 7.6Hz, Ar′-H5′), 6.60 (1H, s, -N-CH-Ar′), 6.88 (1H, d, 7.9 Hz, Ar′-H6′), 7.19-7.32 (5H, m, Ar-H2, 3, 4, 5 & 6 ); FT-IR: 3516 (OH), 2926 (C-H of CH_2_), 1683 (C=O), 1617 (C=N), 1253 (C-O-C) cm^-1^; Anal. Calcd. for C_18_H_18_N_2_O_4_ (326.34): C: 66.25, H: 5.56, N: 8.58. Found: C : 66.22, H : 5.52, N : 5.54; MS: m/z 326 (M^+^), 221 (base peak), 112, 109, 105, 91, 65.

*1-(5-Phenethyl-2-phenyl-1,3,4-oxadiazol-3(2H)-yl)ethanone* (**2d**): White crystals; Yield 60.3%; mp 203-204 °C; ^1^H-NMR δ (ppm): 2.02 (3H, s, -CO-CH_3_), 2.30 (2H, t, 6.4Hz, -CH_2_-C-O-), 2.61 (2H, t, 6.5Hz, Ar-CH_2_), 6.61 (1H, s, -N-CH-Ar′), 7.15-7.29 (10H, m, Ar′ -H2′, 3′, 4′, 5′ & 6′ & Ar -H2, 3, 4, 5 & 6); FT-IR: 1610 (C=N), 2925 (C-H of CH_2_), 1686 (C=O), 1249 (C-O-C) cm^-1^; Anal. Calcd. for C_18_H_18_N_2_O_2_ (294.35): C: 73.45, H: 6.16, N: 9.52. Found: C: 73.42, H: 6.14, N: 9.50; MS: m/z 294 (M^+^), 189 (base peak), 105, 91, 77, 65.

*1-(2-(4-Hydroxyphenyl)-5-phenethyl-1,3,4-oxadiazol-3(2H)-yl)ethanone* (**2e**): Orange crystals; Yield 62.4%; mp 213-214 °C; ^1^H-NMR δ (ppm): 2.05 (3H, s, -CO-CH_3_), 2.35 (2H, t, 6.6Hz, -CH_2_-C-O-), 2.67 (2H, t, 6.5Hz, Ar-CH_2_), 5.26 (1H, s, 4-OH), 6.61 (1H, s, -N-CH-Ar′), 6.68 (2H, d, 7.8Hz, Ar′-H3′ & 5′), 7.03 (2H, d, 7.5Hz, Ar′-H2′ & 6′), 7.16-7.29 (5H, m, Ar-H2, 3, 4, 5 & 6); FT-IR: 3512 (OH), 2920 (C-H of CH_2_), 1680 (C=O), 1613 (C=N), 1249 (C-O-C) cm^-1^; Anal. Calcd. for C_18_H_18_N_2_O_3_ (310.34): C: 69.66, H: 5.85, N: 9.03. Found: C: 69.64, H: 5.82, N: 9.01; MS: m/z 310 (M^+^), 205 (base peak), 112, 105, 93, 91, 65.

## 4. Conclusions 

Both analytical and spectral data (IR, ^1^H-NMR, MS) of all the synthesized compounds were in full agreement with the proposed structure. After comparing the antimicrobial results of compounds **2a-e**, it was concluded that the incorporation of an oxadiazole moiety in phenylpropionyl derivatives enhances their antimicrobial activity and also *para*-substitution in the Ar′ group of the oxadiazoles was found to enhance their potency, especially in compound **2a** and **2b**. Further studies to acquire more information about structure activity relationship are in progress in our laboratory.

## Figures and Tables

**Table 1:** Antimicrobial activity-sensitivity testing of **2a-e**.

Compound No.	Zone of inhibition in mm			
	Antibacterial Activity		Antifungal Activity	
	SA	PA	CA	AF
**2a**	24	24	16	15
**2b**	25	24	15	13
**2c**	23	20	13	12
**2d**	22	23	16	13
**2e**	19	20	16	15
**Ampicillin**	25	24	-	-
**Fluconazole**	-	-	17	16
